# Nighttime warming enhances drought resistance of plant communities in a temperate steppe

**DOI:** 10.1038/srep23267

**Published:** 2016-03-18

**Authors:** Zhongling Yang, Lin Jiang, Fanglong Su, Qian Zhang, Jianyang Xia, Shiqiang Wan

**Affiliations:** 1 State Key Laboratory of Cotton Biology, Key Laboratory of Plant Stress Biology, College of Life Sciences, Henan University, Kaifeng, Henan 475004, China; 2 School of Biology, Georgia Institute of Technology, Atlanta, Georgia 30332, USA; 3 Tiantong National Station of Forest Ecosystem & School of Ecological and Environmental Science, East China Normal University, China

## Abstract

Drought events could have profound influence on plant community structure and ecosystem function, and have subsequent impacts on community stability, but we know little about how different climate warming scenarios affect community resistance and resilience to drought. Combining a daytime and nighttime warming experiment in the temperate steppe of north China with a natural drought event during the study period, we tested how daytime and nighttime warming influences drought resistance and resilience. Our results showed that the semi-arid steppe in north China was resistant to both daytime and nighttime warming, but vulnerable to drought. Nighttime warming, but not daytime warming, enhanced community resistance to drought via stimulating carbon sequestration, whereas neither daytime nor nighttime warming affected community resilience to drought. Large decline in plant community cover, primarily caused by the reduction in the cover of dominant and rare species rather than subordinate species during drought, did not preclude rapid ecosystem recovery. These findings suggest that nighttime warming may facilitate ecosystem sustainability and highlight the need to assess the effects of climate extremes on ecosystem functions at finer temporal resolutions than based on diurnal mean temperature.

Climate warming and changes in precipitation patterns are two major components of climate change, carrying potentially large influences on plant community dynamics and terrestrial carbon cycling^1^,^2^,^3^,^4^. Climate change scenarios, however, are often more complicated than originally thought. For example, nighttime temperature has been found to rise faster than daytime temperature^5^,^6^, rather than a symmetric increase in daytime and nighttime temperature. Precipitation patterns are also expected to be more variable, in addition to predicted changes in average rainfall, resulting in more frequent drought and flooding events^7^,^8^. However, a good understanding of the effects of more realistic scenarios of warming (e.g., asymmetric daytime and nighttime warming) and extreme precipitation events on community and ecosystem properties is still lacking.

Daytime and nighttime warming could influence plant photosynthesis and respiration differently, with potential effects on vegetation productivity and ecosystem carbon processes^9^. For example, Myneni *et al*.^10^ found that increased minimum temperature under nighttime warming enhanced net primary productivity as the result of the prolonged growing season. Consistent with Myneni *et al*.^10^, Wan *et al*.^2^ reported a positive effect of nighttime warming and negative effect of daytime warming on gross ecosystem productivity and net ecosystem productivity. In contrast, Peng *et al*.^11^ reported that vegetation productivity is positively related to maximum temperature in wet and cool ecosystems, and negatively related to maximum temperature in dry temperate regions. Water availability is also one of main drivers of community composition and ecosystem carbon flux. Changes in precipitation may shift species dominance, species distribution^12^,^13^, and grassland ecosystem carbon budgets^14^,^15^. Recent studies have shown that the occurrence of droughts or heatwaves can partially offset carbon sinks or even cause net carbon losses, thereby releasing CO_2_ to the atmosphere^16^,^17^,^18^,^19^. In contrast Griffis *et al*.^20^ found that drought can increase net ecosystem productivity by suppressing ecosystem respiration more than gross ecosystem productivity.

The change in plant community composition and carbon cycles induced by both asymmetrically diurnal warming and extreme drought may have subsequent effect on the resistance of plant production to drought and resilience after the drought events^21^,^22^,^23^,^24^,^25^. Considerable research effort has been devoted to exploring the underlying mechanisms that influence drought resistance and resilience in views of the importance for ecological stability, but a consensus understanding on the key drivers over drought resistance and resilience is still lacking. In particular, the relationship between ecological stability and plant diversity is still controversial. The insurance hypothesis predicts that resistance and resilience would increase with diversity^21^,^26^,^27^,^28^,^29^. However, biomass-dependent hypothesis suggests that drought resistance might be actually determined by plant biomass rather than diversity, with communities with large plant biomass exhibiting increased susceptibility to drought^24^,^29^.

The contribution of dominant species and subordinate species to drought resistance are still debated heatedly^30^. According to the ‘mass ratio’ hypothesis, ecosystem functioning is largely determined by the traits of the dominant species independent of changes in species richness^31^. However, there is growing evidence that subordinate species may also be important^29^,^31^,^32^. Dominant species are expected to respond directly to climate change, and the subordinate species respond not only to climate change directly but also to the changes in dominant species indirectly^3^,^32^. The loss of drought-sensitive dominant species might shift competitive interactions and favour subordinate species with low competitive power^33^. Therefore, subordinate species could thrive under ecological or climate conditions that are unstable^27^.

Here, using a daytime and nighttime warming experiment subjected to a severe natural drought event in a temperate steppe in Northern China, we test three hypotheses: 1) Daytime warming will increase drought resistance and resilience of community cover because of the increased rate of carbon assimilation, whereas nighttime warming will reduce them due to accelerated carbon loss through elevated respiration; 2) The plots with higher diversity would show greater drought resistance and resilience, in a pattern consistent with the insurance hypothesis; 3) Subordinate species may be more resistant to drought than dominant species, due to reduced competition from dominant species during drought. Our results showed that increased carbon sequestration via photosynthesis over-compensation^2^ under nighttime warming enhanced drought resistance of plant community cover. Drought resistance depended on pre-drought plant cover rather than diversity. The reduction in community cover during drought was mainly caused by the drought susceptible dominant species (grass) and rare species (annuals and biennials), but not subordinate species (perennial forbs). Our results supported the hypothesis 3, but refuted the hypotheses 1 and 2.

## Results

### Precipitation

**Figure 1:**
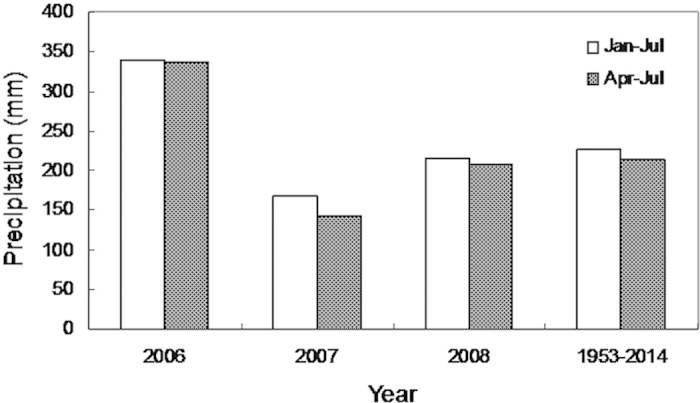
Precipitation from 2006–2008 and mean precipitation from 1953–2014 during Jan-Jul and Apr-Jul at the experimental site.

Jan-Jul and Apr-Jul precipitation in 2007 was 167.3 and 142 mm, respectively, 26% and 33.3% lower than the mean precipitation during the corresponding period from 1953–2014(Fig. 1).

### The community-level response

**Figure 2:**
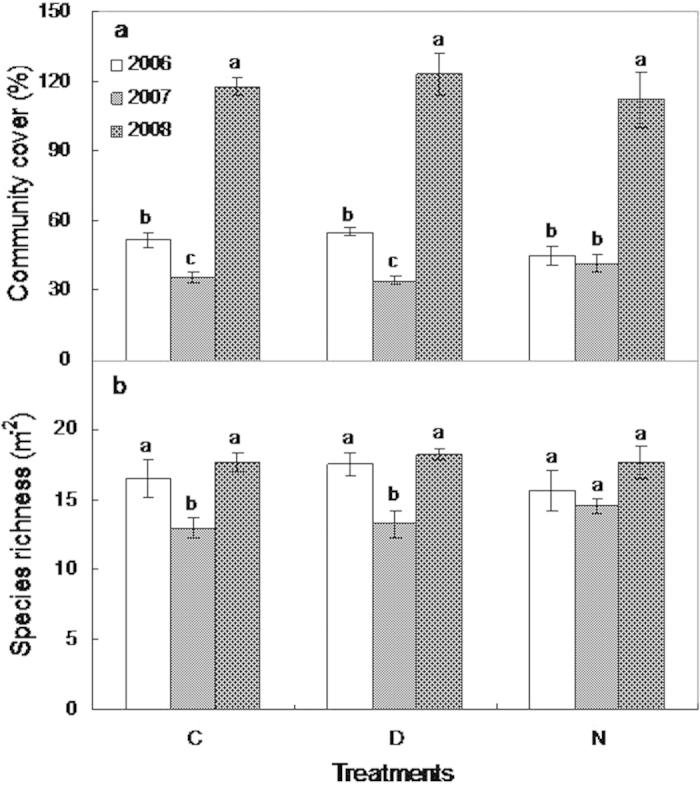
The effects of daytime and nighttime warming on community cover and species richness over the 3 years (2006–2008). C, D, and N represent control, daytime warming, and nighttime warming, respectively. Error bars indicate ± SE.

Neither daytime (F_1,7_ = 0.4, *p* > 0.05) nor nighttime warming (F_1,7_ = 0.1, *p* > 0.05) affected plant community cover (Fig. 2a). Community cover in the control (F_1,7_ = 15.7, *p* = 0.007) and daytime warming (F_1,7_ = 73.9, *p* < 0.001) plots was significantly lower during the drought year (2007) than in the previous year (2006) (Fig. 2a). In contrast, little change in community cover during drought was detected under nighttime warming (F_1,7_ = 0.4, *p* > 0.05). Community cover recovered under all the treatments one year after drought (2008).

No effect of daytime (F_1,7_ = 0.4, *p* > 0.05) or nighttime warming (F_1,7_ = 0.03, *p* > 0.05) on species richness was found (Fig. 2b). Drought reduced species richness by an average of 3.5 (F_1,7_ = 5.1, *p* = 0.065) and 4.25 (F_1,7_ = 11, *p* = 0.002) species m^−2^ in the control and daytime warming plots, respectively. However, drought had no effect on species richness under nighttime warming (F_1,7_ = 0.55, *p* > 0.05; Fig. 2b).

### Ecosystem Carbon flux response

**Figure 3:**
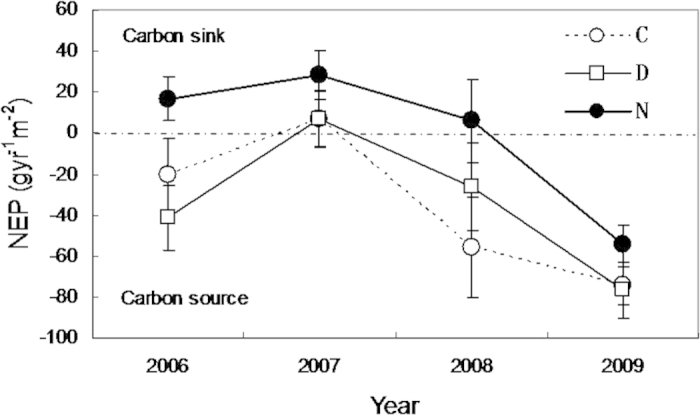
Growing-season (1 May to 31 October) net ecosystem productivity in the four experimental years (2006–2009) were calculated by multiplying daily integrated values of net ecosystem gas exchange by the number of days since the last measuring date. Positive and negative net ecosystem productivity refers to net carbon sink and source, respectively. See Fig. 2 for abbreviations.

Nighttime warming promoted carbon assimilation (F_1,23_ = 4.3, *p* < 0.5; Fig. 3), whereas daytime warming had no effect on carbon flux (F_1,23_ = 0.0, *P* > 0.05; Fig. 3).

### The community composition response

**Figure 4:**
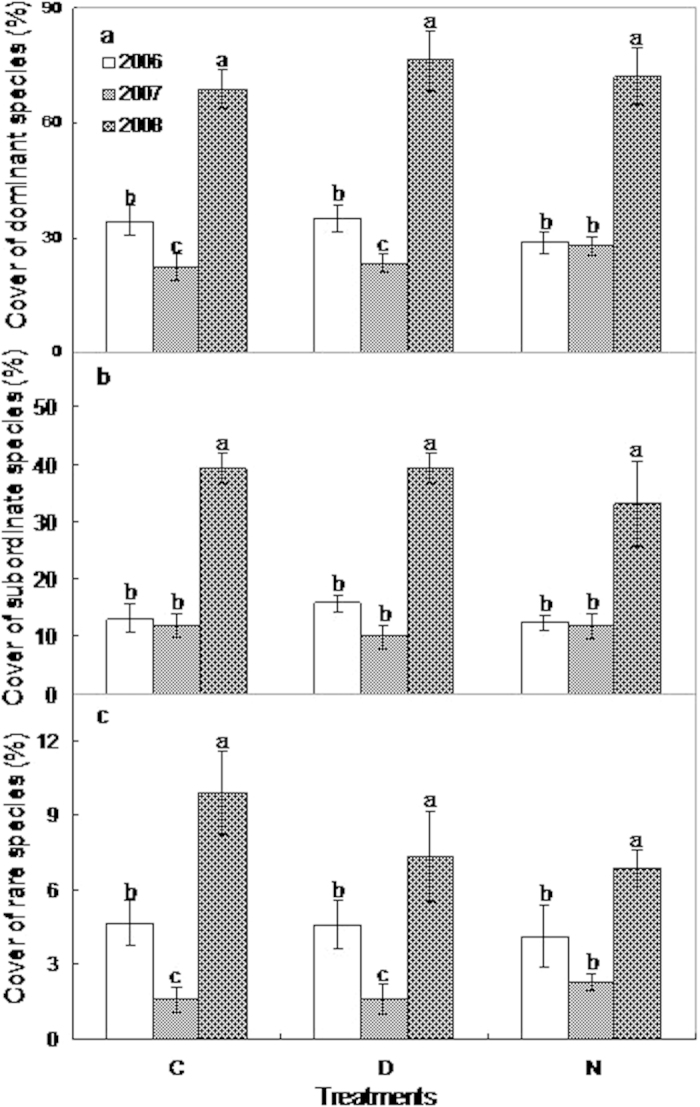
The effects of daytime and nighttime warming on the dominant, subordinate and rare species cover across the three years (2006–2008). Error bars indicate ± SE. See Fig. 2 for abbreviations. See Fig. 2 for abbreviations.

Neither daytime nor nighttime warming affected the cover of dominant, subordinate, or rare species from 2006–2008 (Fig. 4). Drought reduced dominant species cover by 35.3% and 48.8% under control (F_1,7_ = 5.8, *p* = 0.05) and daytime warming (F_1,7_ = 7.1, *p* = 0.04), respectively, but not under nighttime warming (F_1,7_ = 0.05, *p* > 0.05; Fig. 4a). Subordinate species cover was not affected by drought under any of the three treatments (Fig. 4b). Drought reduced rare species cover by 66% (F_1,7_ = 8.5, *p* = 0.03) and 65.6% in the control and daytime warming plots (F_1,7_ = 7, *p* = 0.04), respectively, but not in the nighttime warming plots (F_1,7_ = 2, *p* > 0.05; Fig. 4c).

**Figure 5:**
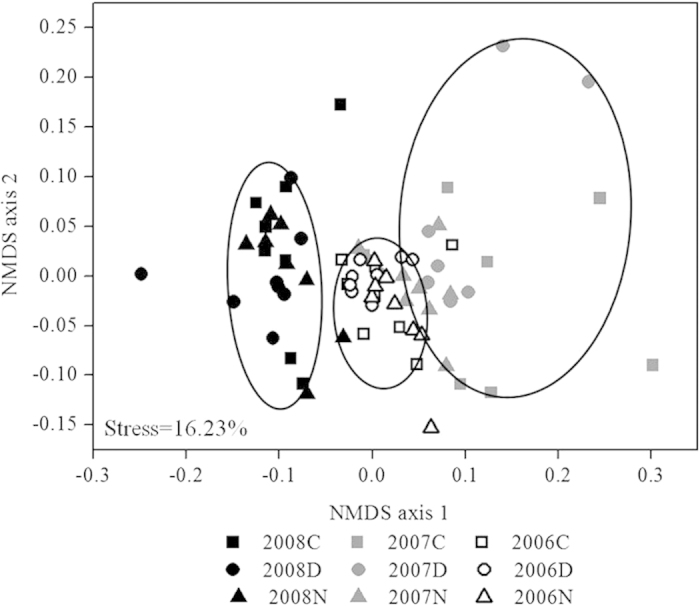
The effects year, daytime and nighttime warming on community composition according to non-metric multidimensional scaling ordination of Bray-Curtis distance matrixes. See Fig. 2 for abbreviations.

Non-metric multidimensional scaling ordination of Bray-Curtis distance matrixes of species cover from 2006–2008 data showed that neither daytime nor nighttime warming changed community composition. However, community composition varied substantially across the three years (stress = 16.23%, Fig. 5). Two-way permutational ANOVA showed that year had a significant effect on composition (F = 5.26, *p* < 0.001, *r*^2^ = 0.13), whereas warming had no significant effect (F = 2.43, *p* > 0.05, *r*^2^ = 0.06).

### Drought resistance and resilience

**Figure 6:**
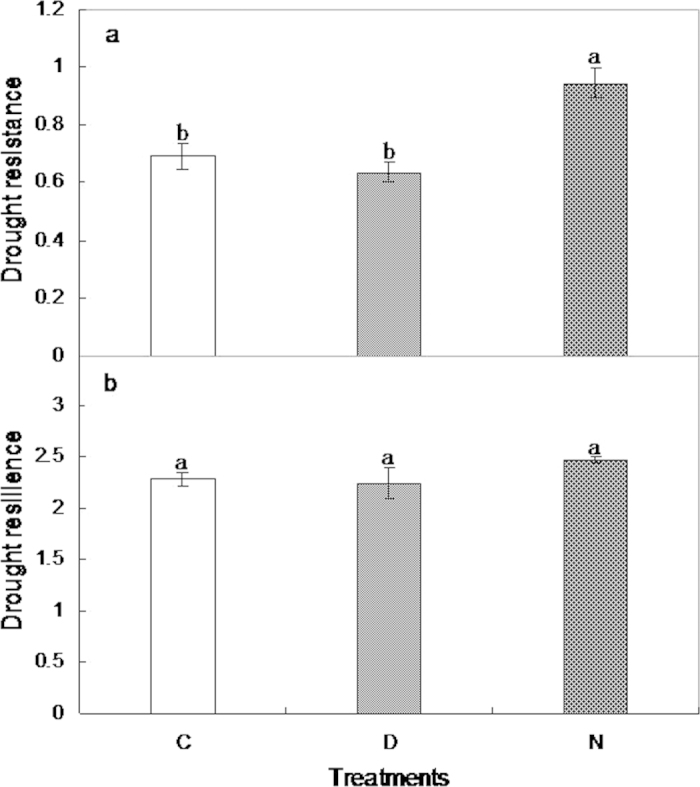
The effects of daytime and nighttime warming on drought resistance and resilience. Error bars indicate ± SE. See Fig. 2 for abbreviations.

Nighttime warming enhanced community resistance to drought (F_1,7_ = 6.6, *p* < 0.05; Fig. 6a) by 36.1%, but did not affect community resilience after drought due to the little change of community cover during drought (F_1,7_ = 3.0, *p* > 0.05; Fig. 6b). In contrast, no effect of daytime warming on community resistance (F_1,7_ = 0.5, *p* > 0.05; Fig. 6a) or resilience (F_1,7_ = 0.03, *p* > 0.05; Fig. 6b) was detected.

### Factors affecting drought resistance

Community resistance decreased with increasing pre-drought community cover (F_1,15_ = 11.5, *p* = 0.004, *r*^*2*^ = 0.45; Table 1), but was unaffected by pre-drought species richness (F_1,15_ = 0.1, *P* > 0.05, *r*^*2*^ = 0). Further analysis revealed a negative correlation between drought resistance and dominant species cover in 2006 (F_1,15_ = 9.7, *p* = 0.008, *r*^*2*^ = 0.41; Table 1).

## Discussion

In this study, we examined how daytime and nighttime warming influenced the response of a semiarid steppe ecosystem to extreme drought in North China. Our result showed that temperate steppe in north China is resistant to warming, but vulnerable to drought. Community cover declined with the decreasing cover of dominant and rare species rather than subordinate species during drought. Despite low resistance to drought, this ecosystem exhibited high resilience, and community cover has complete recovery one-year after drought. Contrasting with daytime warming, the increased net ecosystem productivity under nighttime warming enhanced drought resistance. These findings suggest that the enhanced ecosystem sustainability under nighttime warming could help provide more reliable goods and service for human beings under the intensified global climate change scenarios.

The low resistance to drought observed in this study is consistent with the findings of Tilman and Downing^21^ and Hoover *et al*.^33^, who found a rapid loss in ecosystem function with drought, but conflicts with findings of Evans *et al*.^34^ who reported substantial resistance to drought in a semiarid grassland. Contrasting sensitivities of different grassland ecosystems to drought can be ascribed to drought intensity, duration, and timing^33^.The ecological response to an extreme drought event observed in our study can be explained by the timing of the drought. The high sensitivity of productivity and seedling survival to water requirement in the earlier stages of plant growth would decrease ANPP and maximize the plant mortality, given extremely low spring rainfall^35^,^36^.

Plant communities produced less biomass during drought when containing higher biomass of dominant species prior to drought. One possible reason could be that dominant species with high stature (Fig. S1) consume more soil water via evapotranspiration^29^, and lead to serious water limitation. This finding provides further support for the biomass-dependent hypothesis as proposed by Wang *et al*.^29^. The lack of plant diversity effect on drought resistance found here is inconsistent with the results in several manipulative experiments, which found positive effect of diversity on drought resistance^26^,^37^,^38^. One possible reason could be that the randomly assembled plant communities in most manipulative experiments may neglect the competitive hierarchy amongst species in natural communities^29^.

The decrease in the abundance of dominant and rare species (specifically grasses, annuals, and biennials, Fig. S2) and the compensatory increase in subordinate species (perennial forbs, whose absolute cover remained constant, but relative cover increased during drought, Fig. S2) in response to drought indicate that subordinate species may not be most resistant to drought, but may benefit most from increasing drought stress and reduced competition from dominant species. This finding is consistent with that of Mariotte^30^ who found that the importance of subordinate species to ecosystem functions may be strengthened in a fluctuating environment, but disagree with the mass ratio hypothesis which suggests that ecosystem functioning is determined by the traits of the dominant species^31^. Drought stress might have depleted shallower soil moisture layers, leading to decreased abundance of dominant (grass) and rare species (annuals and biennials). Thus a drought tolerance strategy fails for the species (grasses, annuals and biennials plants) with shallower roots, while a drought avoidance strategy allows persistence of the perennial forbs with deeper root^33^.

The reduction in ecosystem productivity during drought does not preclude rapid ecosystem recovery. The immediate recovery observed in this study disagrees with previous reports of prolonged effects of a single drought event on production^21^,^39^,^40^, and also contrasts with the legacy effects of drought observed by others^41^,^42^ who have found that drought-induced structural changes in the vegetation influence production in the subsequent year. The potential underlying mechanisms that determine the rapid resilience in our system could be ascribed to several aspects. First, the abundant rainfall in 2008 (412 mm) provides the necessary condition for rapid vegetation recovery. Second, many plant species in the temperate steppe in northern China are perennials with clonal growth. Thus the damage of drought on roots is relatively small compared with the damage on aboveground parts of plants (Fig. S3), leading to quick recovery of vegetation after drought. Third, soil seed bank in the temperate steppe may promote recovery indirectly by increasing the probability of species recruitment^43^,^44^. There were 92.3 ± 17.8 seeding germinations in 1 m^2^ area, which provides another important guarantee for the fast recovery of vegetation. Fourth, according to the stress-gradient hypothesis^45^, competition should be more important under less stressful conditions and species biomass in this type of communities usually shows negative correlations. By contrast, facilitation becomes more important in harsh environments and positive correlations between species will be more common^45^,^46^,^47^,^48^,^49^. The reduced complementary effect and the enhanced compensatory effect after drought in the present study (Fig. S4) imply that competition dominates the system and the vegetation is recovering according to succession theory^48^. Finally, mineral and organic substrates tend to accumulate during dry periods while there is little nutrient demand, leading to an excess of mineralization during the early phases of the wet cycle, which would enhance productivity and promote recovery^49^.

The increased drought resistance under nighttime warming can be explained by the leaf-level carbon cycle. Increased carbon sequestration under nighttime warming via photosynthesis over-compensation (nighttime warming enhanced consumption of carbohydrates in the leaves, and consequently stimulated plant photosynthesis in the next day)^2^ would outweight the negative effects of drought, and finally result in enhanced drought resistance under nighttime warming. Neither changes in drought resistance nor drought resilience were observed under daytime warming, despite the fact that daytime warming influenced soil moisture more strongly than nighttime warming. One possible explanation for the lack of daytime warming effect is that the influence of daytime warming on community cover and plant growth is much smaller than that of drought. This is partially supported by the finding of Shi *et al*.^50^ that plant communities in tallgrass prairie were resistant to experimental warming in the first seven years (2000–2006), but vulnerable in an extreme wet year (2007). Another alternative explanation is that the positive effects of daytime warming on plant growth via extending the length of growing seasons^11^,^51^ may have been mitigated by the negative effect of increased water limitation under daytime warming^1^,^51^, resulting in a neutral change of drought resistance or resilience under daytime warming.

## Materials and Methods

### Study site

The study was carried out at a semiarid temperate steppe (42°02′N, 116°17′E, 1324 m a.s.l) in Inner Mongolia, China. Mean annual temperature is 2.1 °C, ranging from −17.5 °C in January to 18.9 °C in July. Mean annual precipitation over the previous 50 years is 383 mm, with most falling in the growing season (from May to October). According to the Chinese classification, the sandy soil at the study site is chestnut, with mean bulk density of 1.31 g cm^−3^ and pH of 7.7.

The dominant species in this temperate steppe, which has relatively low aboveground primary productivity (approx. 100–200 gm^−2^ yr^−1^), are perennial plants, including *Artimesia frigida, Stipa capillata, Leymus chinensis, Cleistogenes caespitosa*, and *Potentilla acaulis*.

### Experimental design

The experiment used a random block design with three treatments, including the control (C), daytime warming (D; 6:00 am–6:00 pm, local time), and nighttime warming (N; 6:00 pm–6:00 am); each treatment had four replicates^6^. The plot size is 3 × 4 m with a 3-m distance between any two adjacent plots. All the warmed plots were heated by MSR-2420 infrared radiators (Kalglo Electronics Inc, Bethlehem, PA, USA) suspended 2.25 m above the ground from March 16 to November 15. In order to simulate the shading effects, we also placed a“dummy”heater with the same shape and size as the infrared heater in each of the control plots. All the heaters under the warming treatments were set at an electrical power output of approximately 1,600 W^51^. The warming treatment started from 23 April, 2006 and run continuously the first two years (2006 and 2007) and from March 16 to November 15 since the year of 2008.

### Soil temperature (ST) and moisture (SM)

Soil temperature at 10 cm depth was measured with the thermocouple probe (Li-8100-201, Li-Cor, Inc., Lincoln, NE, USA) three times a month from May to October. SM (0–10 cm) was measured using a portable soil moisture device (Diviner 2000, Sentek Pty Ltd, Balmain, Australia) weekly during the growing season. Based on our previous study, daytime and nighttime warming increased daily mean soil temperature by 0.71 and 0.78 °C, respectively. Daytime warming reduced soil moisture by 0.88 V/V%, whereas nighttime warming had little effect on soil moisture^51^,^52^.

### Ecosystem Carbon fluxes

Ecosystem carbon fluxes were measured using a transparent sampling chamber (50 cm length × 50 cm width × 50 cm height) attached to the LI-6400 (LI-COR 6400, Li-Cor, Lincoln, NE, USA) for all the subplots. Net ecosystem exchange and ecosystem respiration were measured three times per month over the growing seasons. During each measurement, the chamber was sealed with the surface of aluminum frame which was installed into soil 2–3 cm, and two small fans ran continuously to mix the air inside the sampling chamber. The chamber was opened for 1–2 minutes following the measurements of net ecosystem exchange, and then covered with an opaque cloth for ecosystem respiration measurement. The rates of ecosystem CO_2_ fluxes were measured three times a month from May to October. Each measurement event lasted 24 hour, with the carbon flux data recorded every three hours.

### Vegetation monitoring

From 2006–2008, we estimated plant cover in two 1 × 1 m permanent quadrats in each plot in August, when plant biomass reached its peak level^3^. Directly estimating biomass in a permanent quadrat would bring disturbance to the biomass of the next year and therefore was not used. We note that plant cover is often a reasonable proxy of plant biomass, and this proxy has been used in many other studies^36^,^53^,^54^,^55^,^56^. The plant cover of the two quadrats in each plot was averaged for statistical analyses. In each quadrat, species richness was recorded. We made sure to limit this estimate to include species that actually rooted within the quadrat, excluding species with parts overhanging the plot only. The cover of each species in each quadrat was estimated using a canopy interception technique based on 100 equally distributed grids (10 × 10 cm). The cover of each species was visually estimated in all the grids and summed for the quadrat. Plants were divided into five different functional groups according to growth form, including perennial rhizome grass, perennial bunchgrasses, perennial forbs, shrubs and semi-shrubs, and annuals and biennials^53^. Within each quadrat, the plant cover of each functional group or the whole community was calculated as the summed cover of plant species belonging to the functional group or the community. Summed cover of all species may exceed 100%.

### Defining dominant, subordinate and transient species

We also classified species according to their relative abundances. A species was classified as ‘dominant’ if its mean relative abundance in the control plots exceeded 5%, as ‘subordinate’ if its mean relative abundance was between 1% and 5%, and as ‘rare’ if its mean relative abundance was <1%^30^,^54^. This classification regime yielded 5 dominant species, 11 subordinate species, and 32 rare species. The names of the dominant and non-dominant species and their functional group identities were included in Table S1. The temperate steppe is co-dominated by shrubs and semi-shrubs (mainly *A. frigida*) and grass (including perennial bunchgrasses and perennial rhizome grass, mainly *S. capillata, A. cristatum* and *Cleistogenes caespitosa*). Perennial forb is the most species-rich group, which includes most of the subordinate and rare species. Annuals and biennials is the least abundant functional group and contribute little to community biomass (see Table S1 for details).

## Calculations

In the present study, drought resistance was quantified as the ratio of community cover in a drought year (2007) to community cover in the previous non-drought year (2006), and drought resilience was quantified as the ratio of community cover in the first year after drought (2008) to the cover in the previous year of drought (2006)^24^,^29^. Proportional values were chosen over absolute values because they have the advantage of being scale-free^29^. Note that although we did not manipulate drought directly, our approach provides a way to investigate the importance of natural drought events^55^,^56^,^57^.

### Statistical analysis

One-way ANOVA was performed to test the effects of daytime and nighttime warming on drought resistance, resilience, and net ecosystem productivity. Also, we use one-way ANOVA to test for the difference in community cover, species richness, the cover of dominant, subordinate and rare species in the controls, daytime and nighttime warming in drought year (2007) with those in the pre-drought year (2006). LSD post-hoc comparisons were used to determine the significance of differences between different levels of warming. Species cover from 2006–2008 under control, daytime and nighttime warming was used to examine the changes of community composition by non-metric multidimensional scaling ordination of Bray-Curtis distance matrixes.

In order to test whether pre-drought’s community cover, species richness, and the cover of dominant, subordinate, and rare species affect drought resistance, multiple regression analyses were used to test the relationships between drought resistance and these variables. Two-way permutational ANOVA was used to investigate year and treatment effects on community composition. All analyses were performed with the SPSS15.0 (SPSS institute, 2008) and R v.2.14.0 (R Development Core Team 2011).

## Additional Information

**How to cite this article**: Yang, Z. *et al*. Nighttime warming enhances drought resistance of plant communities in a temperate steppe. *Sci. Rep.* **6**, 23267; doi: 10.1038/srep23267 (2016).

## Supplementary Material

Supplementary Information

## Figures and Tables

**Table 1:** Relationship between drought resistance and five parameters which hypothesized as the drivers of drought resistance. *F* value and *r*^*2*^ were showed.

Variables	Drought resistance	
	***F***_***1,15***_	***r***^***2***^
Community cover in 2006	11.5^**^	0.45
Species richness in 2006	0.1^NS^	0.0
Dominant species cover in 2006	9.7^**^	0.41
Subordinate species cover in 2006	0.5^NS^	0.04
Rare species cover in 2006	0.0^NS^	0.0
