# Supplementary material for: Nighttime warming enhances drought resistance of plant communities in a temperate steppe
Source: Sci Rep. 2016 Mar 18;6:23267. doi: 10.1038/srep23267 (PMC4796875; doi:10.1038/srep23267)
Supplement: Supplementary Information [file srep23267-s1.doc]

**Supporting information**

**Title:** Nighttime warming enhances drought resistance of plant communities in a temperate steppe

**Zhongling Yang^1^, Lin Jiang^2^, Fanglong Su^1^, Qian Zhang^1^, Jianyang Xia^3^, and Shiqiang Wan^1^***

Table S1. The name of dominant (D), subordinate (S), rare species (R) and the division of functional groups were showed. Nomenclature follows the editorial committee of Chinese plant records (1998).

| **Perennial Forbs (PF)** | **Semi-Shrubs (SS)** | **Grass** | **Annuals and Biennials (AB)** |
| --- | --- | --- | --- |
| *Heteropappus altaicus* (S) | *Artemisia frigida*(D) | *Stipa* *capillata*(D) | *Salsola collina*(R) |
| *Potentilla tanacetifolia* (S) | *Lespedeza bicolor*(R) | *Leymus chinensis*(D) | *Chamaerhodos erecta*(R) |
| *Thalictrum alpinum* (R) |  | *Cleistogenes songorica*(D) | [*Chenopodium aristatum*](http://frps.eflora.cn/frps/Chenopodium aristatum)(R) |
| *Gueldenstaedtia verna* (S) |  | *Achnatherum sibiricum*(R) | *Chenopodium glaucum*(R) |
| *Phlomis umbrosa*(S) |  | *Koeleria cristata*(R) | *Dontostemon dentatus*(R) |
| *Potentilla acaulis*(D) |  | *Botriochloa ischaemum*(R) | *Silene conoidea*(R) |
| *Potentilla bifurca*(S) |  | *Agropyron cristatum*(S) | *Setaria* *viridis*(R) |
| *Astragalus galactites*(S) |  | *Poa subfastigiata*(R) | *Suaeda glauca*(R) |
| *Allium sikkimense*(R) |  |  |  |
| *Potentilla multifida*(R) |  |  |  |
| *Medicago ruthenica*(S) |  |  |  |
| *Sibbaldia procumbens*(S) |  |  |  |
| *Carex lanceolata*(S) |  |  |  |
| *Gentiana squarrosa*(R) |  |  |  |
| *Bupleurum scorzonerifolium*(R) |  |  |  |
| *Allium neriniflorum*(R) |  |  |  |
| *Dianthus chinensis*(R) |  |  |  |
| *Allium ramosum*(R) |  |  |  |
| *Astragalus scaberrimus*(S) |  |  |  |
| *Iris tenuifolia*(R) |  |  |  |
| *Artemisia scoparia*(R) |  |  |  |
| *Allium bidentatum*(R) |  |  |  |
| *Artemisia pubescens*(R) |  |  |  |
| *Erodium stephanianum*(R) |  |  |  |
| [*Gentiana dahurica*](http://frps.eflora.cn/frps/Gentiana dahurica)(R) |  |  |  |
| *Saposhnikovia divaricata*(R) |  |  |  |
| *Astragalus melilotoides*(R) |  |  |  |
| *Cymbaria dahurica*(R) |  |  |  |
| *Ixeridium graminifolium*(R) |  |  |  |
| *Potentilla betonicaefolia*(R) |  |  |  |

**Figure S1**.The effects of daytime and nighttime warming on the cover of species height ≥ 20cm and cover of species height < 20cm over time. Error bars indicate ± SE. See Fig. 2 for abbreviations

Drought reduced the cumulative cover of species height < 20 cm by 32.1% in daytime warming (F_1,15_=17.1, *p*=0.001), but not in others treatments (Fig. S1a). Drought reduced the cumulative cover of species height≥20 cm by 49.5% in control (F_1,15_=9.1, *p*=0.009), 75.7% in daytime warming (F_1,15_=17.2, *p*=0.001), and 80.8% in nighttime warming (F_1,15_=35, *p*<0.001; Fig. S1b).

**Figure S2**. The effects of daytime and nighttime warming on the cover of grass, semi-shrubs, and perennial forbs over the 3 years. Error bars indicate ± SE. See Fig. 2 for abbreviations.

Drought reduced grass cover by 78.2% (F_1,7_=14.8, *p*<0.01), 53.0% (F_1,7_=6.9, *p*<0.05), and 45.4% (F_1,7_=9.3, *p*<0.05; Fig. S2a) under control, daytime, and nighttime warming, respectively. Drought declined semi-shrubs abundance by 48.9% under daytime warming (F_1,7_=9.0, *p*<0.05; Fig. S2b), but not under the other two treatments. Perennial forb was not affected by drought under any of the three treatments (Fig. S2c).

**Figure S3**. The ratio of root biomass to ANPP in the 1×1 m permanent quadrats across the 3 years. See Fig. 2 for abbreviations. Error bars indicate ± SE

**Figure S4**. Species cover’correlation matrix among 33 species pairs in all the treatments in 2007 and 2008. Correlation analyses showed that negative correlations pairs reduced while positive correlation pairs increased from 2007 to 2008 under control, daytime, and nighttime warming. See Fig. 2 for abbreviations.

The result showed that the positive correlation pairs reduced, while the negative correlation pairs increased after drought under control, daytime warming, and nighttime warming, suggesting that complementary effects decreased, however, compensatory effects increased from 2007 (a drought year) to 2008. The result implies that the system is in a steady recovery phase in terms of stress-gradient hypothesis (SGH) and succession theory. Most species pairs did not show any statistically significant correlation, indicating that a statistical averaging effect might have been operating at the species level.

**Figure S5**. The relationship between community cover and annual primary productivity in control plots across year of 2006-2008.

**Figure S6**. Mean temperature during the Jan-Dec from 2006 to 2008 at the experimental site.
